# The Hippo Pathway Controls a Switch between Retinal Progenitor Cell Proliferation and Photoreceptor Cell Differentiation in Zebrafish

**DOI:** 10.1371/journal.pone.0097365

**Published:** 2014-05-14

**Authors:** Yoichi Asaoka, Shoji Hata, Misako Namae, Makoto Furutani-Seiki, Hiroshi Nishina

**Affiliations:** 1 Department of Developmental and Regenerative Biology, Medical Research Institute, Tokyo Medical and Dental University, Tokyo, Japan; 2 Centre for Regenerative Medicine, Department of Biology and Biochemistry, University of Bath, Claverton Down, Bath, United Kingdom; Toho University School of Medicine, Japan

## Abstract

The precise regulation of numbers and types of neurons through control of cell cycle exit and terminal differentiation is an essential aspect of neurogenesis. The Hippo signaling pathway has recently been identified as playing a crucial role in promoting cell cycle exit and terminal differentiation in multiple types of stem cells, including in retinal progenitor cells. When Hippo signaling is activated, the core Mst1/2 kinases activate the Lats1/2 kinases, which in turn phosphorylate and inhibit the transcriptional cofactor Yap. During mouse retinogenesis, overexpression of Yap prolongs progenitor cell proliferation, whereas inhibition of Yap decreases this proliferation and promotes retinal cell differentiation. However, to date, it remains unknown how the Hippo pathway affects the differentiation of distinct neuronal cell types such as photoreceptor cells. In this study, we investigated whether Hippo signaling regulates retinogenesis during early zebrafish development. Knockdown of zebrafish *mst2* induced early embryonic defects, including altered retinal pigmentation and morphogenesis. Similar abnormal retinal phenotypes were observed in zebrafish embryos injected with a constitutively active form of *yap* [(*yap (5SA)*]. Loss of Yap’s TEAD-binding domain, two WW domains, or transcription activation domain attenuated the retinal abnormalities induced by *yap (5SA)*, indicating that all of these domains contribute to normal retinal development. Remarkably, *yap (5SA)*-expressing zebrafish embryos displayed decreased expression of transcription factors such as *otx5* and *crx,* which orchestrate photoreceptor cell differentiation by activating the expression of *rhodopsin* and other photoreceptor cell genes. Co-immunoprecipitation experiments revealed that Rx1 is a novel interacting partner of Yap that regulates photoreceptor cell differentiation. Our results suggest that Yap suppresses the differentiation of photoreceptor cells from retinal progenitor cells by repressing Rx1-mediated transactivation of photoreceptor cell genes during zebrafish retinogenesis.

## Introduction

In the vertebrate embryonic nervous system, multipotent neural progenitor cells proliferate and differentiate into diverse neuronal and glial cell types that eventually build up functional neural circuits such as the retina [1], [2]. The retina is a delicate multilayered neural epithelium composed of six types of neurons and one major type of glial cell [3]. During the course of retinal development, retinal progenitor cells (RPCs) either continue to proliferate or exit mitosis and differentiate into various neuronal cell types. This process is tightly regulated and ensures that the proper numbers and types of differentiated cells needed to assemble a functional retinal circuitry are produced [1], [2]. A fundamental mystery in retinal development has been the identity of the molecular mechanism controlling the developmental switch between RPC self-renewal and differentiation. Although rodent models have provided valuable insights into the molecular basis of vertebrate retinal development [4], the zebrafish (*Danio rerio*) is a good alternative in which to seek the definitive answer to this question [5], [6]. Fertilized zebrafish eggs rapidly develop *ex utero* into transparent embryos, facilitating retinal observations and experimental manipulations such as morpholino knockdown and the use of transgenic technology. In addition, aspects of retinal morphogenesis and histology, as well as the molecular components governing retinal development, are highly conserved between zebrafish and mammals.

The FGF, Shh, Wnt and Notch signaling pathways have all been identified as affecting retinal cell proliferation and differentiation [7]. For instance, the Notch pathway normally suppresses photoreceptor cell production in the mammalian retina, whereas inhibition of Notch signaling enhances the expression of the *Otx2* and *Crx* genes, which encode transcription factors (TFs) expressed exclusively in photoreceptor cells [8]–[10]. Another important pathway recently shown to be involved in regulating the balance between RPC maintenance and differentiation is the Hippo signaling cascade [11]. Hippo signaling plays fundamental roles in organ size control, stem cell maintenance, and progenitor differentiation in a variety of tissues, including the central nervous system (CNS) [12]–[14]. When activated by a developmental cue, the Hippo core Mst1/2 kinases activate the Lats1/2 kinases, which in turn phosphorylate and negatively regulate the transcriptional cofactor Yap. Control of Yap in this way modulates the transcription of many genes required for tissue-specific cell differentiation [15].

The importance of the Hippo pathway in retinogenesis has been revealed by studies in mice and zebrafish. For example, gene knockout mice lacking Sav1, a component of the Hippo pathway, showed impaired organization of the retinal epithelium during neurogenesis [16]. In a different study, forced expression of Yap in the developing mouse retina led to RPC proliferation and inhibition of retinal differentiation [11]. In zebrafish, knockdown of Yap decreased progenitor cell populations in the CNS, including in the eye [17]. These observations suggest that the Hippo pathway is essential for controlling the balance of self-renewal and differentiation in developing RPCs. However, the precise molecular mechanism by which the Hippo pathway regulates the differentiation of specific types of retinal neurons has remained obscure. In particular, there is little information on the target retinal TF(s) activated downstream of the Hippo-Yap pathway. In this study, we show that the TF Rx1, a novel interacting partner of Yap, is a missing piece of this puzzle and contributes to retinal photoreceptor cell differentiation regulated by the Hippo-Yap pathway. We propose a model in which Yap regulates the timing of photoreceptor cell differentiation by suppressing Rx1-mediated transactivation of the *otx, crx* and *rhodopsin* genes.

## Results

### Mst2 is Required for Early Embryogenesis in Zebrafish

**Figure 1:**
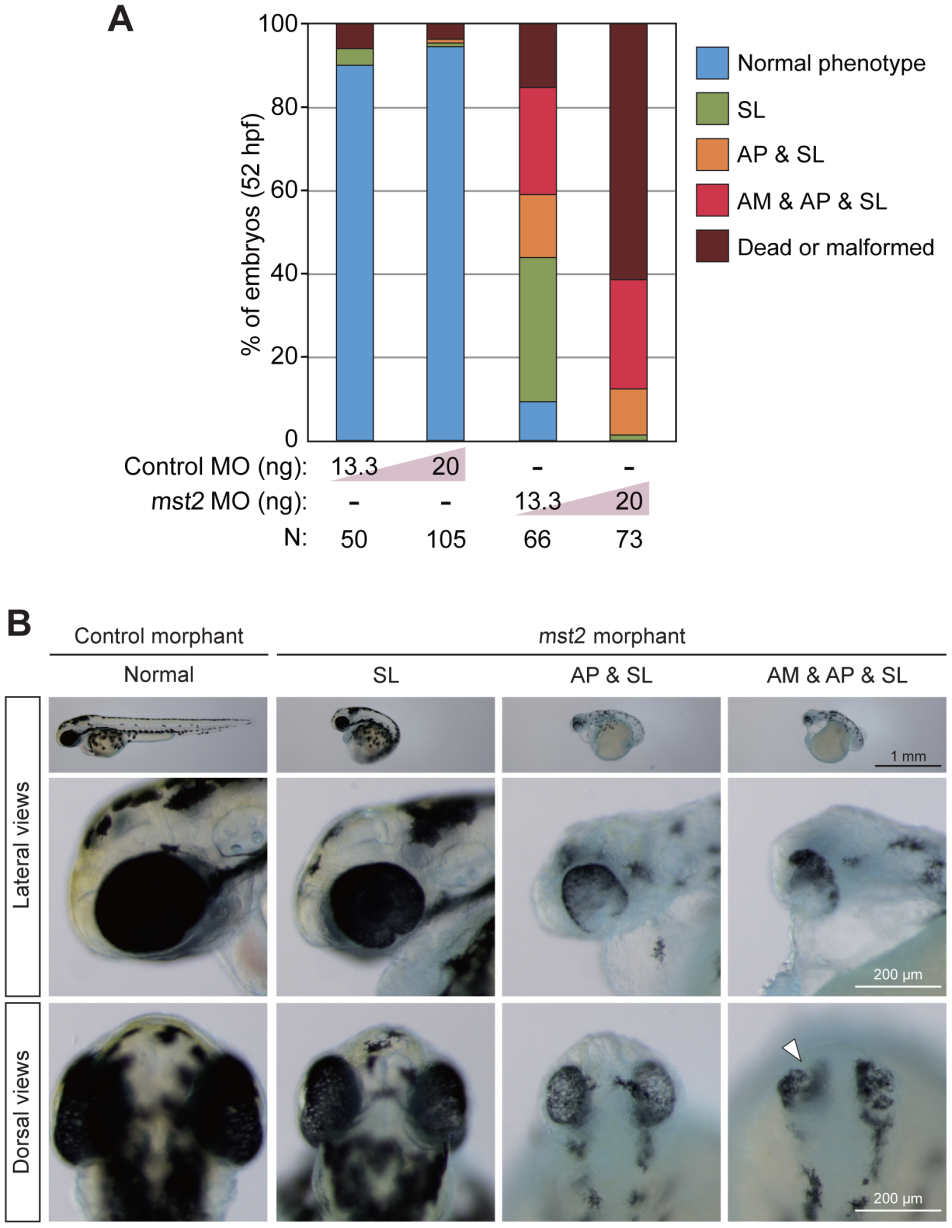
Mst2 is essential for early zebrafish embryogenesis. (A) Early developmental abnormalities of *mst2* morphants. Control or *mst2* morpholino (MO) at the indicated dose was injected into zebrafish embryos and phenotypes were analyzed at 52 hpf. Embryos were classified into five color categories on the basis of their phenotypes: blue, normal embryos; green, short body length (SL); orange, abnormal eye pigmentation (AP) accompanied by SL; red, abnormal eye morphology (AM) plus AP plus SL; and brown, dead or malformed embryos. Results are presented as the percentage of the total number of embryos examined (N). (B) Representative control and *mst2* morphants at 52 hpf. Embryos were injected with control MO (13.3 ng) or *mst2* MO (13.3 ng). Top panels, lateral views of whole embryos. Middle panels, higher magnification images of the head regions of the embryos in the top panels. Bottom panels, dorsal views of the head regions of the embryos in the top panels. (The head is at the top of each panel.) White arrowhead, representative area of AM.

To unravel the role of Hippo signaling in early zebrafish development, we first examined whether zebrafish *mst* functions during early embryogenesis. We performed BLAST searches with human *MST1* and *MST2* genes to predict the sequence of zebrafish *mst* cDNA and found that the zebrafish has only one *mst2* ortholog. The predicted amino acid sequence of the protein encoded by the zebrafish *mst2* gene is approximately 90% identical to the sequences of the human and mouse Mst2 proteins, and contains the evolutionarily conserved autophosphorylation site and SARAH domain that are important for Mst activation (Fig. S1A). A phylogenetic analysis confirmed that the zebrafish *mst2* gene was clustered with those of several vertebrate species, including teleosts (Fig. S1B). To determine the functionality of the zebrafish *mst2* gene, we performed a morpholino (MO)-mediated loss-of-function analysis. Zebrafish embryos treated with *mst2* MO (*mst2* morphants) showed a range of abnormal phenotypes at 52 hours post-fertilization (hpf), from short body length (SL) to abnormal eye pigmentation (AP) and abnormal eye morphology (AM) (Fig. 1A and 1B). RT-PCR analysis confirmed that microinjection of *mst2* MO had effectively prevented correct splicing of the targeted pre-mRNA (Fig. S1C and S1D). These results demonstrate that Mst2 plays a critical role in early zebrafish embryogenesis.

### Yap Activity has Important Effects on Early Zebrafish Development

**Figure 2:**
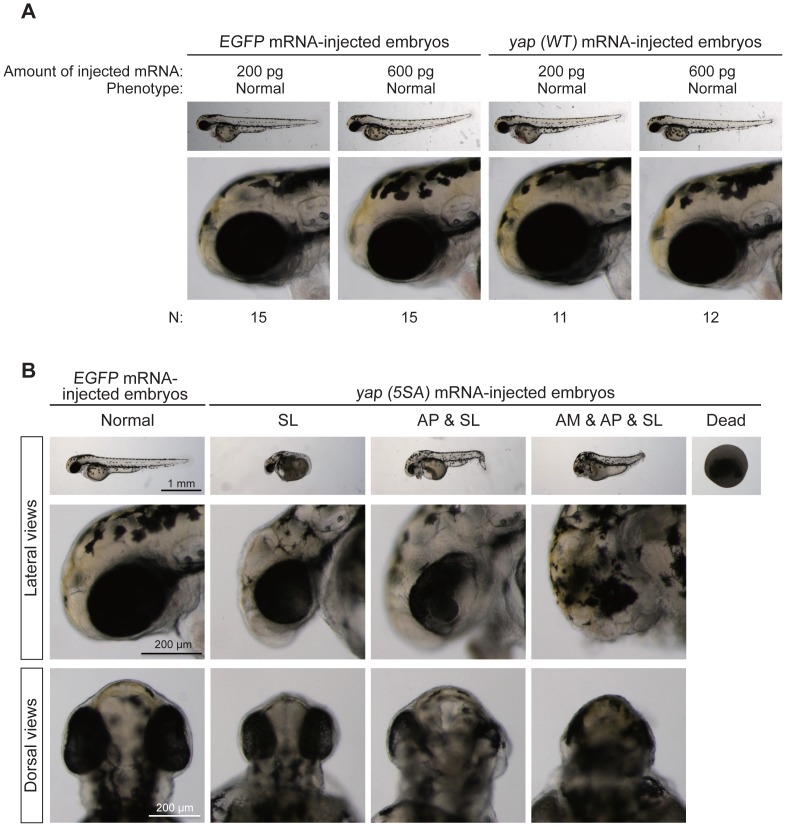
Forced expression of mRNA encoding constitutively active *yap* alters early zebrafish embryogenesis. (A) Representative images of *EGFP* mRNA-injected (control) or *yap (WT)* mRNA-injected zebrafish embryos at 52–54 hpf. Top panels, lateral views of whole embryos. Bottom panels, higher magnification images of the head regions of the embryos in the top panels. N, total number of embryos examined. Embryos injected with either *yap (WT)* mRNA or *EGFP* mRNA had normal phenotypes. (B) Representative images of *EGFP* mRNA-injected (control) or *yap (5SA)* mRNA-injected zebrafish embryos at 48 hpf. Embryos injected with *Yap (5SA)* mRNA (10 pg) showed the same spectrum of abnormal phenotypes as *mst2* morphants. Data are presented as for Fig. 1B.

Since Yap is a key effector molecule downstream of the Hippo signaling pathway [12], [13], we determined whether overexpression of *yap* induced morphological phenotypes similar to those observed in *mst2* morphants. The amino acid sequence of the Yap protein in the small fish medaka is 85% identical to that of the zebrafish Yap protein and contains the five sites normally phosphorylated by Lats in vertebrate Yap (Fig. S2A). It is now well established that the Hippo pathway regulates Yap’s phosphorylation, subcellular localization, and transcriptional coactivator activity, and that this control mechanism is evolutionarily conserved among vertebrates [18]. Some post-translational modifications of Yap, such as its acetylation, are also highly conserved among vertebrates [19]. These observations gave us confidence that medaka Yap (WT) would be functionally comparable with zebrafish Yap (WT) in our experiments. In addition, we generated a constitutively active form of medaka Yap called Yap (5SA) in which the five sites normally targeted by Hippo pathway-dependent phosphorylation were mutated to alanine [20]. Normal zebrafish embryos that were injected with *in vitro*-transcribed medaka *yap (WT)* mRNA were indistinguishable from *EGFP* mRNA-injected control embryos during the first 2 days of development (Fig. 2A). However, by 48 hpf, embryos that had been injected with constitutively active *yap (5SA)* mRNA exhibited the same range of abnormal phenotypes (SL, AP and AM) as seen in the *mst2* morphants (Fig. 2B). These observations indicate that Yap acts downstream of Mst2 to influence early zebrafish development.

### The TEAD-binding, WW and Transcription Activation Domains of Yap are Required for Normal Zebrafish Embryogenesis

**Figure 3:**
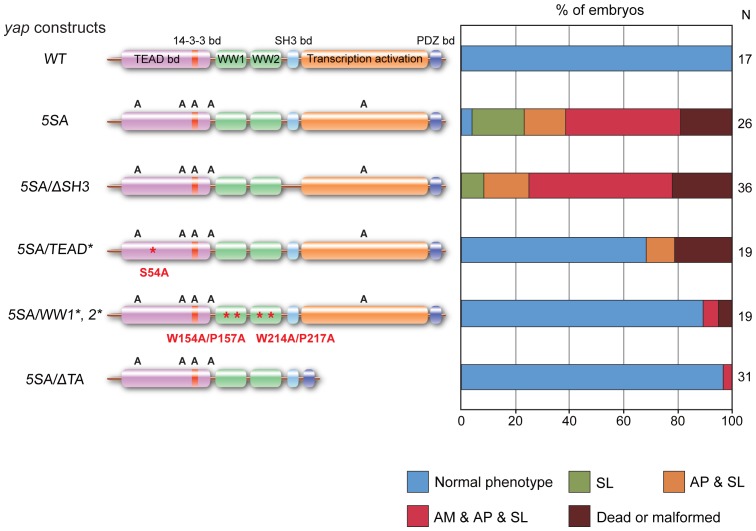
The TEAD-binding, WW and transcription activation domains of Yap contribute to early zebrafish development. Left panel, schematic illustration of constructs of Yap (WT), Yap (5SA), and the indicated variants with deletion (Δ) or mutation (*) of the indicated domains. Specific amino acid alterations are indicated. A, Lats phosphorylation site replaced by an alanine. *In vitro*-synthesized mRNAs (10 pg) derived from these constructs were injected into zebrafish embryos and phenotypes were quantified as shown in the right panel. Color classification is as for Fig. 1A. Results are presented as the percentage of the total number of embryos examined (N).

To define which functional domains of Yap are important for early zebrafish development, we created a series of *yap (5SA)* constructs bearing mutations or deletions inactivating specific Yap domains (Fig. 3). Injection of *yap (5SA)* mRNA led to the same range of developmental defects as presented in Fig. 2B (SL, 19%; AP+SL, 15%; AM+AP+SL, 42%; normal phenotype, 4%; N = 26). Similar results were observed for embryos injected with *yap (5SA)* mRNA missing its SH3-binding domain [*yap (5SA/ΔSH3)*]. In contrast, expression of a *yap (5SA)* mRNA with a defect in the TEAD-binding domain [yap (5SA/TEAD^*^)] reduced the frequency of abnormal phenotypes (AP+SL, 11%; normal phenotype, 68%; N = 19). In addition, the majority of embryos injected with *yap (5SA)* mRNA mutated in both the WW1 and WW2 domains [yap (5SA/WW1^*^, 2^*^)] exhibited a normal phenotype (AM+AP+SL, 5%; normal phenotype, 89%; N = 19). Finally, almost all embryos injected with *yap (5SA)* mRNA missing its transcription activation domain [*yap (5SA/ΔTA)*] showed a normal phenotype (AM+AP+SL, 3%; normal phenotype, 97%; N = 31). Taken together, these observations demonstrate that overexpression of the TEAD-binding, WW and transcription activation domains of Yap can alter early zebrafish development, and that these domains are therefore critical for normal zebrafish morphogenesis.

### Yap Activity Plays a Direct Role in Zebrafish Retinogenesis

**Figure 4:**
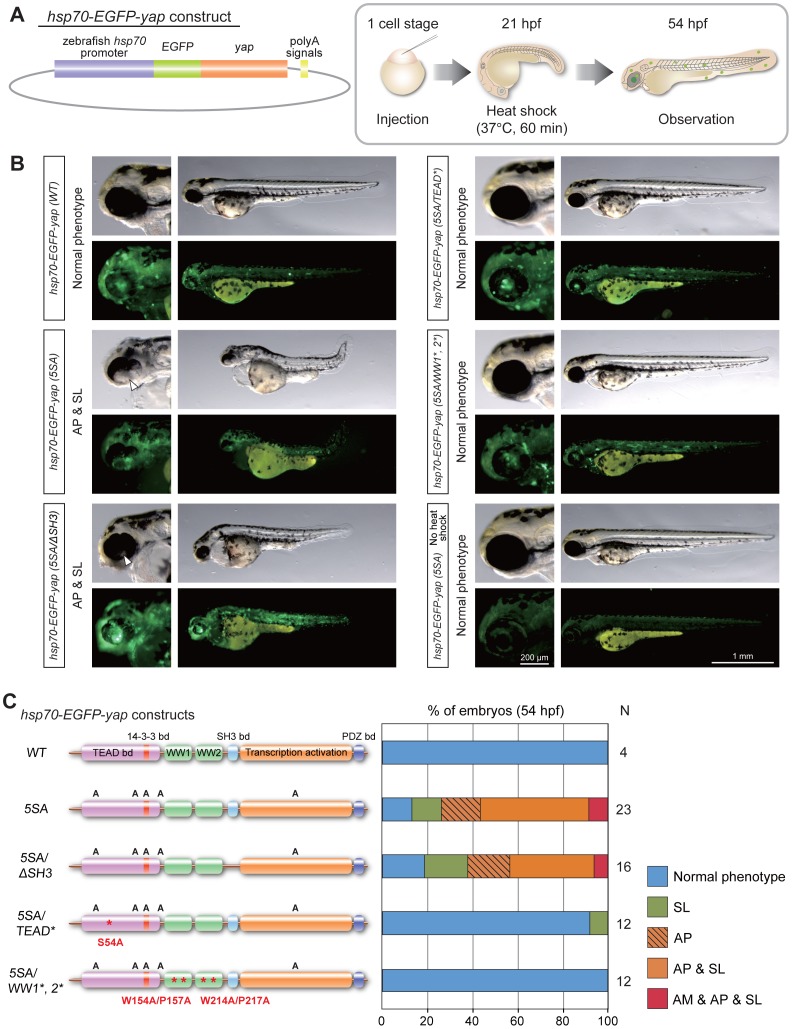
Yap is directly involved in zebrafish retinogenesis. (A) Schematic illustration of the base *hsp70*-*EGFP*-*yap* construct (left panel) and the procedure for the heat shock experiment (right panel). Zebrafish embryos at the one-cell stage were injected with plasmid DNA containing the heat shock promoter constructs indicated in (B). At 21 hpf, injected embryos were immersed in a 37°C water bath for 1 h to apply heat shock and thus induce expression of EGFP-fused Yap. At 54 hpf, EGFP-expressing embryos were isolated and classified on the basis of their phenotypic features. (B) Representative images of the embryos in (A) that were injected with heat shock promoter constructs as indicated on the left side of panels. For each column, top right panels show lateral views of whole embryos, top left panels show higher magnification images of the head regions of the embryos, and bottom panels are fluorescent images of the corresponding top panels. White arrowheads, areas of AP. (C) Quantification of phenotypes of the embryos injected with heat shock promoter constructs in (A, B) as analyzed at 54 hpf. Color classification is as for Fig. 1A except that the phenotype of AP alone is indicated by striped orange shading. Results are presented as the percentage of the total number of embryos examined (N).

Our experiments in Figure 3 showed that injection of *yap (5SA)* mRNA caused abnormal retinal development and body axis malformation. However, it was not clear whether the retinal abnormality was a primary consequence of Yap hyperactivation or a secondary effect caused by the failure in body axis formation. To distinguish between these possibilities, we examined in detail the timing of the emergence of the SL phenotype in *yap (5SA)* mRNA-injected zebrafish embryos. Overexpression of *yap (5SA)* mRNA induced no obvious defects during gastrulation or anterior-posterior axis formation (Fig. S2B), consistent with previous work [21]. After gastrulation, however, the SL phenotype became apparent at 18–21 hpf (Fig. S2C), indicating that increased Yap activity affects the elongation of the body axis during the segmentation period. To minimize the effects of body axis malformation, we generated a *yap (5SA)* construct under the control of the zebrafish heat shock-inducible promoter *hsp70* [*hsp70*-*EGFP*-*yap (5SA)*] [22], and induced *yap (5SA)* expression only after 21 hpf (Fig. 4A). Whereas injection alone of *hsp70*-*EGFP*-*yap (5SA)* induced no phenotypic alterations, heat shock applied at 21 hpf after injection of *hsp70*-*EGFP*-*yap (5SA)* gave rise to abnormal retinal phenotypes (AM and/or AP) (Fig. 4B and 4C). It is noteworthy that, although many embryos also exhibited the SL phenotype (AP+SL, 48%; AM+AP+SL, 9%; N = 23), a sizable proportion showed only an abnormal retinal phenotype (AP, 17%; N = 23). These results support our hypothesis that Yap activity has a direct impact on retinal development.

**Figure 5:**
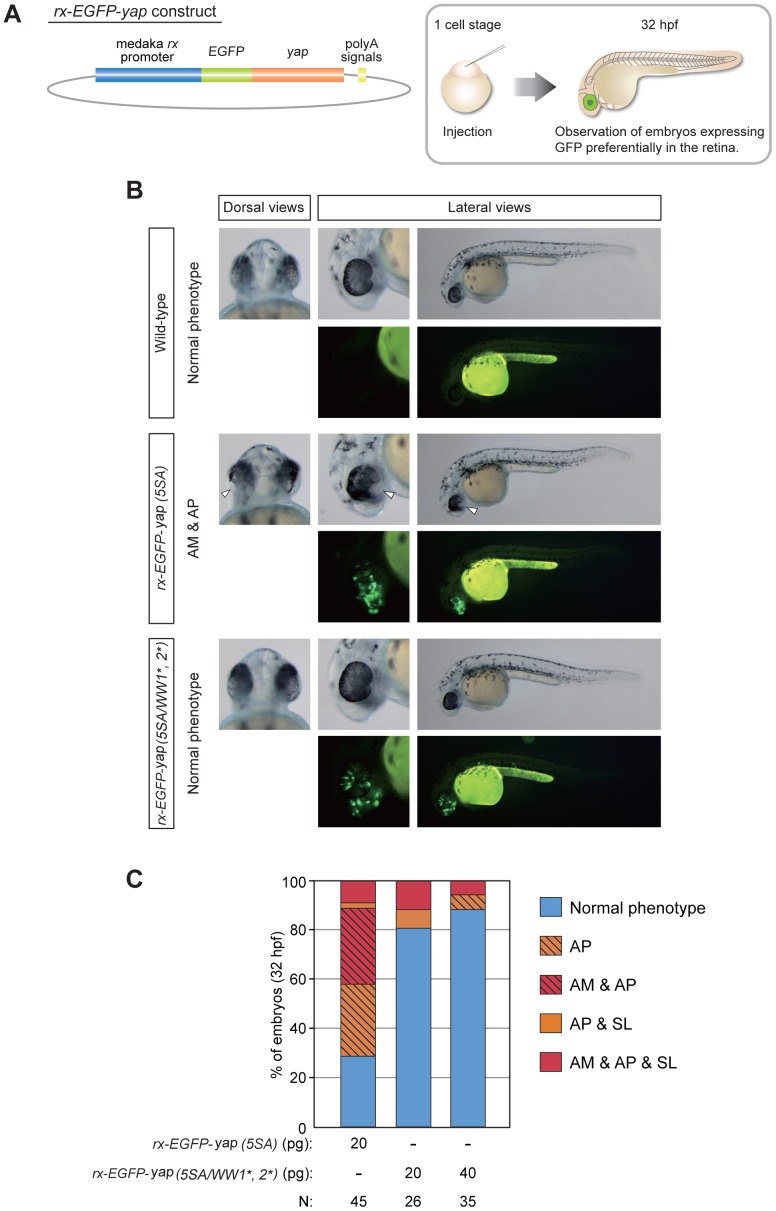
Retina-specific expression of *yap (5SA)* induces retinogenesis defects without affecting body axis formation. (A) Schematic illustration of the base *rx*-*EGFP*-*yap* construct (left panel) and the procedure for the experiment (right panel). Zebrafish embryos at the one-cell stage were injected with plasmid DNA containing the *rx* promoter constructs indicated in (B). (B) Representative dorsal and lateral views of the embryos in (A) that were injected with *rx* promoter constructs as indicated on the left side of panels. Data are presented as for Fig. 4B. White arrowheads, areas of AM plus AP. (C) Quantification of phenotypes of the embryos injected with *rx* promoter constructs in (A, B) as analyzed at 32 hpf. Color classification is as for Fig. 4C except that the phenotype of AM plus AP is indicated by striped red shading. Results are presented as the percentage of the total number of embryos examined (N). Note that expression of *yap (5SA)* variants mutated in both the WW1 and WW2 domains prevented the appearance of abnormal eye phenotypes.

To achieve retina-specific expression of Yap, we generated a construct containing the upstream region (including the promoter) of the medaka *rx3* gene [*rx*-*EGFP*-*yap (5SA)*]. Injection of this plasmid into zebrafish embryos resulted in expression of *yap (5SA)* preferentially in the retina (Fig. 5A). Expression of *rx*-*EGFP*-*yap (5SA)* gave rise to abnormal eye phenotypes (AM and/or AP) in about 60% of injected embryos (AP, 29%; AM+AP, 31%; N = 45), with no detectable effect on body axis (Fig. 5B and 5C). Conversely, expression of *yap (5SA)* variants mutated in both WW domains [*rx*-EGFP-yap (5SA/WW1^*^, 2^*^)] prevented the appearance of abnormal eye phenotypes. These data demonstrate that the two WW domains of Yap mediate activity that directly affects zebrafish retinogenesis.

### Retinal Photoreceptor Genes are Downregulated in *yap (5SA)*-expressing Embryos

**Figure 6:**
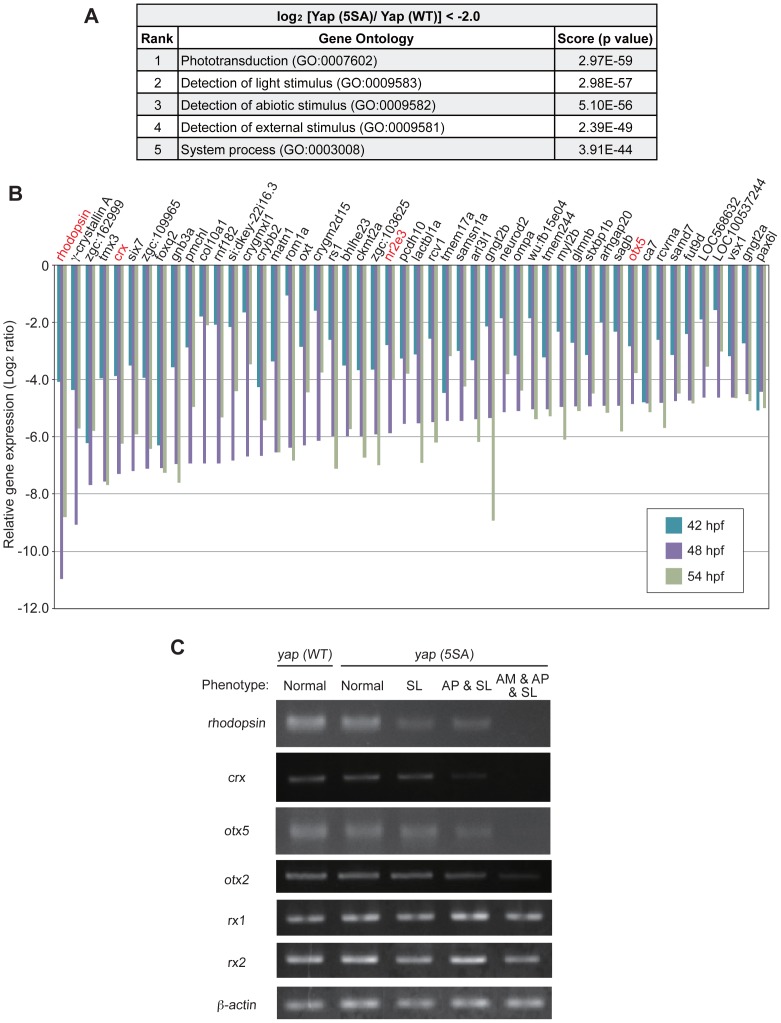
*Yap (5SA)* mRNA-injected embryos exhibit dramatic downregulation of retinal photoreceptor genes. (A) The top five GO categories for genes downregulated by over 4.0-fold in *yap (5SA)*-expressing embryos at 48 hpf as determined by microarray analysis. (B) A summary of microarray results for the top 50 downregulated genes in the *yap (5SA)*-expressing embryos in (A) compared with *yap (WT)*-expressing embryos at 42, 48 and 54 hpf. The expression levels of genes in the *yap (5SA)*-injected embryos are shown as Log_2_ (fold change) values relative to *yap (WT)*-injected embryos. The order of the genes is based on expression levels detected at 48 hpf. Red lettering indicates retinal photoreceptor genes whose expression was severely decreased in *yap (5SA)*-injected embryos. (C) RT-PCR analysis of mRNA expression of the indicated retinal genes in zebrafish embryos injected with *yap (WT)* or *yap (5SA)* mRNA and examined at 48 hpf. β-actin, loading control. *Yap (5SA)*-expressing embryos are grouped by abnormal phenotype, as indicated. Results are representative of two independent experiments.

To conduct a comprehensive survey of transcriptional targets activated downstream of Hippo-Yap signaling during early zebrafish development, we employed a microarray approach and compared genome-wide transcriptomes between *yap (WT)-* and *yap (5SA)*-expressing embryos at three developmental stages (42, 48 and 54 hpf). Gene ontology (GO) analysis revealed that the top two GO categories for genes showing a >4.0-fold decrease in expression in *yap (5SA)*-expressing embryos at each stage were “phototransduction” and “detection of light stimulus” (Fig. 6A). Strikingly, the retinal photoreceptor gene *rhodopsin* was the gene most downregulated in *yap (5SA)*-expressing embryos compared to *yap (WT)*-expressing embryos (Fig. 6B). This remarkable decrease was 17.0-fold at 42 hpf, an enormous 1,974-fold at 48 hpf, and 449-fold at 54 hpf. Moreover, we found that expression levels of genes encoding photoreceptor TFs such as *crx*, *nr2e3* and *otx5*, which are required for *rhodopsin* transcription [23]–[25], were greatly reduced in *yap (5SA)*-injected embryos (decreased by 157-, 58.8-, and 29.1-fold, respectively, at 48 hpf) (Fig. 6B). These results indicate that the expression of *yap (5SA)* mRNA affects the transcription of retinal photoreceptor genes.

To confirm Yap’s influence on retinal gene expression, we carried out a detailed RT-PCR analysis of mRNA levels in *yap (5SA)*-injected embryos and *mst2* morphants. We found that mRNA levels of *otx2, otx5, crx* and *rhodopsin* were all dramatically downregulated in *yap (5SA)*-injected embryos at 48 hpf compared to *yap (WT)*-injected embryos (Fig. 6C). *Mst2* morphants also displayed decreased mRNA expression of the *otx2*, *crx*, and *rhodopsin* genes (Fig. S3A and S3B). Lastly, because Rx is known to be an upstream transactivator that regulates *otx2* and *rhodopsin* expression in mice and *Xenopus* [9], [26], we examined whether expression of the *rx1* and *rx2* genes was reduced in *yap (5SA)*-expressing embryos. Interestingly, levels of *rx1* and *rx2* mRNAs in *yap (5SA)*-injected embryos were comparable to those in *yap (WT)*-injected embryos (Fig. 6C). These results suggest that Yap activity affects zebrafish retinogenesis via transcriptional regulation of photoreceptor genes acting downstream of the *rx* genes.

### The Photoreceptor Cell Differentiation Factor Rx1 is a Novel Interacting Partner of Yap

**Figure 7:**
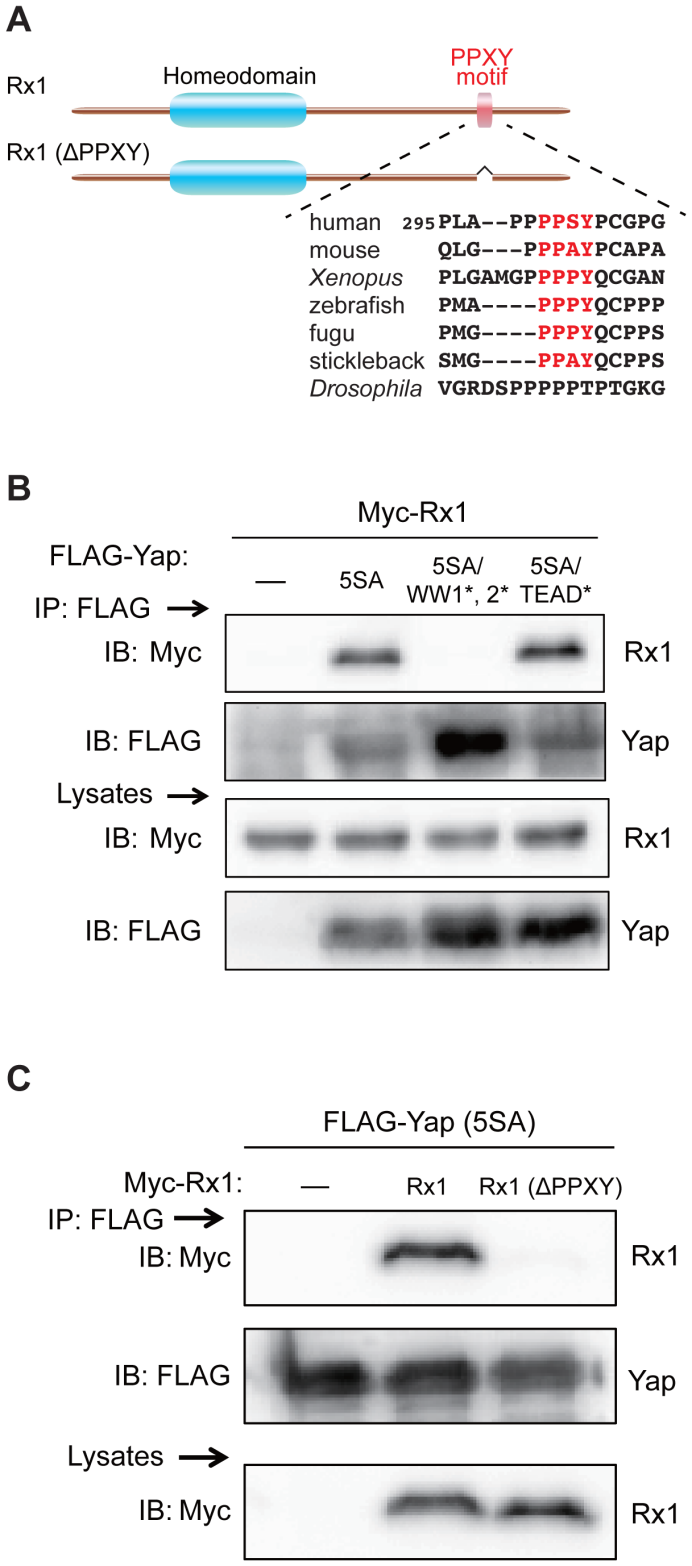
The WW domains of Yap interact with the PPXY motif of Rx1. (A) Schematic illustration of the zebrafish Rx1 and Rx1 (ΔPPXY) constructs. A partial amino acid sequence including the PPXY motif of zebrafish Rx1 was aligned with the sequences of Rx from the indicated species. The PPXY motif (red lettering) is highly conserved among vertebrates. A detailed alignment of Rx family proteins can be found in Fig. S5A. (B) Co-immunoprecipitation analysis of HEK293T cells transiently expressing Myc-Rx1 that were co-transfected with empty vector (–), or vector expressing Yap (5SA), Yap (5SA/WW1^*^, 2^*^) or Yap (5SA/TEAD^*^). Lysates were immunoprecipitated (IP) with anti-FLAG Ab to isolate Yap, followed by Western blotting (WB) with anti-Myc Ab to detect Myc-Rx1 (top), or with anti-FLAG Ab to detect FLAG-Yap (middle). (C) Co-immunoprecipitation analysis of HEK293T cells transiently expressing FLAG-Yap (5SA) that were co-transfected with empty vector (–), or vector expressing Rx1 or Rx1 (ΔPPXY). Lysates were IP’d using anti-FLAG Ab and subjected to WB with anti-Myc Ab to detect Rx1 (top), and with anti-FLAG Ab to detect Yap (middle). Bottom, WB analysis of total cell lysate using anti-Myc Ab to detect Rx1.

The above microarray and RT-PCR analyses suggested that activated Yap might suppress photoreceptor cell differentiation through interactions with TF(s) acting upstream of *otx, crx* and *rhodopsin*. We investigated Rx1 as a candidate TF in this context because zebrafish Rx1 reportedly plays a prominent role in the regulation of retinal photoreceptor differentiation [27]. Intriguingly, we found that zebrafish Rx1 contains an evolutionarily conserved PPXY motif that interacts with Yap’s WW domains (Fig. 7A), whereas none of the other three photoreceptor TFs examined (Otx2, Otx5 and Crx) contains a PPXY motif. This observation prompted us to use co-immunoprecipitation analysis to investigate whether Yap and Rx1 could physically interact with each other in cells. Myc-Rx1 was co-expressed with FLAG-Yap (5SA), FLAG-Yap (5SA/WW1^*^, 2^*^), or FLAG-Yap (5SA/TEAD^*^) in HEK293T cells, and cell lysates were subjected to immunoprecipitation with anti-FLAG antibody. We observed that Myc-Rx1 successfully co-immunoprecipitated with either FLAG-Yap (5SA) or FLAG-Yap (5SA/TEAD^*^) but not with FLAG-Yap (5SA/WW1^*^, 2^*^) (Fig. 7B). These results demonstrate that Rx1 can indeed interact with Yap, and that this interaction is mediated by Yap’s two WW domains. We also co-expressed FLAG-Yap (5SA) with Myc-Rx1 missing its PPXY motif [Myc-Rx1 (ΔPPXY)] in HEK293T cells and subjected cell lysates to immunoprecipitation with anti-FLAG antibody. Myc-Rx1 (ΔPPXY) did not co-immunoprecipitate with FLAG-Yap (5SA) (Fig. 7C), indicating that the PPXY motif of Rx1 is essential for its interaction with Yap. These data identify the photoreceptor cell differentiation factor Rx1 as a novel interacting partner of Yap, and suggest that Yap may be crucial for coordinating the timing of the terminal differentiation of photoreceptor neurons by suppressing the transcription of the *otx, crx* and *rhodopsin* genes.

## Discussion

In this study, we examined the role of Hippo-Yap signaling during zebrafish retinogenesis by carrying out an *in vivo* analysis. We demonstrated that knockdown of Mst2 or forced expression of *yap (5SA)* not only disrupts normal embryogenesis as a whole but has specific detrimental effects on retinal pigmentation, eye morphology, and the expression of retinal photoreceptor genes. With respect to embryogenesis, the SL phenotype we observed in our *yap (5SA)* mRNA-injected embryos at 18–21 hpf (Fig. S2C) is similar to that of morphants created in a previous study by knockdown of the zebrafish *yap* gene [17], [28]. These latter morphants exhibited a shortened body axis and elevated expression of the somite marker *myoD* during somitogenesis. Our findings thus provide additional evidence that strict control of the activity and localization of Yap is essential for normal somitogenesis during the earliest stages of embryogenesis. Moreover, our data show that Hippo-Yap signaling acts at a later developmental stage as a crucial switch governing retinogenesis.

A key result of our paper is that Yap and the retina-specific TF Rx1 physically interact with each other through Yap’s WW domains and Rx1’s PPXY motif. Fig. S4 illustrates our proposed model for the bifunctional involvement of Hippo-Yap signaling in determining RPC proliferation versus photoreceptor cell differentiation. When the Hippo pathway is inactive, Yap is activated and associates with TEAD to help drive expression of proliferation-related genes. Simultaneously, activated Yap binds to Rx1 and attenuates its transactivation of photoreceptor genes. The result is the expansion of RPCs and the suppression of photoreceptor cell differentiation. However, when the Hippo pathway is activated by a developmental cue, Yap activation is blocked and the expression of photoreceptor genes is upregulated, promoting the differentiation of mature photoreceptor cells. Thus, in this model, Hippo-Yap signaling is the key molecular mechanism governing the decision of an RPC to self-renew or differentiate.

In *Drosophila*, Hippo is the homolog of mammalian Mst2. In the *Drosophila* eye, Hippo is involved in post-mitotic fate-determining events such as photoreceptor subtype specification [29]. It is conceivable that the primary role of Mst2 in the developing eye is evolutionarily conserved among vertebrate species. In our study of MO-mediated knockdown of zebrafish *mst2,* we showed that this gene is essential for retinal photoreceptor differentiation (Fig. 1A, 1B and S3). In *Xenopus*, Nejigane *et al.* [2013] carried out a loss-of-function analysis of *mst1/2* and found that *mst2* morphants displayed morphogenetic defects, including abnormally small eyes [30]. However, it has been difficult to determine the separate physiological functions of the mammalian *Mst1* and *Mst2* genes during retinal development due to their overlapping tissue expression and functional redundancy. For example, both the *Mst1* KO and *Mst2* KO single null mutant strains are viable and develop normally, suggesting a substantial functional overlap between these two paralogs [31]. Further functional analysis of *Mst1/2* genes in other vertebrates should help to reveal more about the possible evolutionary diversion of Mst1 and Mst2.

Previous studies have implicated Hippo signaling in ocular development [11], [16], [17], [30]. For example, Zhang *et al.* observed that forced expression of Yap in mouse retina prevented proneural bHLH proteins from inducing cell cycle exit, whereas inhibition of Yap decreased RPC proliferation and increased retinal cell differentiation [11]. However, few studies have focused on the molecular mechanism(s) by which Hippo-Yap signaling regulates the differentiation of specific neuronal subtypes such as photoreceptor cells. In our study, we demonstrated that at least three photoreceptor TFs (Otx2, Otx5 and Crx) are activated downstream of Hippo signaling (Fig. 6C and S3). In addition, we discovered that Rx1 is a novel interacting partner of Yap (Fig. 7B), a finding that supplies a missing piece of the puzzle concerning the molecular basis of Hippo-Yap-mediated effects on photoreceptor cell differentiation. In mouse studies, Rx is essential for *otx2* transactivation in the embryonic retina [9]. In *Xenopus* retina, Rx reportedly plays a role in the transcriptional regulation of other retinal photoreceptor genes, such as *rhodopsin* and *red cone opsin* [26]. In zebrafish, Rx1 is required for photoreceptor differentiation [27]. These previous results, together with our present study, support the idea that the timing of activation of both the Rx1-*otx/crx* and Rx1-*rhodopsin* transcriptional cascades is regulated by the Hippo-Yap pathway during zebrafish photoreceptor development.

Our mutational analysis of the Yap (5SA) protein demonstrated that Yap’s TEAD-binding, WW, and transcription activation domains all play a pivotal role in the regulation of retinogenesis (Fig. 3). TEAD family members have previously been shown to be critical partners of Yap in regulating neural progenitor cells. For example, Yap functions through TEAD family members to control the proliferation of progenitors in the chicken spinal cord [32]. In the *Xenopus* neural plate, Yap and TEAD1 cooperate to expand neural progenitors and directly regulate *pax3* expression [21]. Our study therefore provides more evidence that the precise regulation of Yap-TEAD interaction is important for maintaining normal neurogenesis. In addition to TEAD family members, PPXY motif-containing TFs, such as ErbB4, p73 and RUNX2, have been shown to interact with Yap via its WW domains [33]–[35]. For instance, Yap suppresses RUNX2-dependent transcriptional activation of the *osteocalcin* gene promoter [36]. Our study identifies zebrafish Rx1 as a novel photoreceptor differentiation factor, and shows that Rx1’s PPXY motif interacts with the WW domains of Yap. This result is consistent with previous observations that many protein interactions associated with Hippo-Yap signaling rely on the binding of a protein’s PPXY motif to Yap’s WW domains [37]–[39]. We postulate that Yap functions as a bifunctional transcriptional cofactor by using its TEAD-binding or WW domains; i.e., Yap co-activates the proliferation of RPCs induced by TEAD family members, but also co-represses retinal photoreceptor differentiation through interaction of its WW domains with Rx1 (Fig. S4).

It is worth noting that the zebrafish genome contains additional PPXY motif-containing retinal TFs, including ROR members and Nrl (Fig. S5B and S5C); these proteins could also be potential Yap targets. In particular, zebrafish RORα and RORβ possess a PPXY motif that is highly conserved among vertebrate species (Fig. S5B). Furthermore, RORα and RORβ are known to be crucial for photoreceptor cell differentiation because they directly regulate multiple photoreceptor genes [40]–[42]. Further analyses of TFs expressed in vertebrate photoreceptor tissues should help to evaluate the general role of Yap in photoreceptor cell differentiation.

Yap and its paralogous coactivator TAZ are central nuclear effectors of Hippo signaling and play critical roles in early development [43]. In most vertebrates, Yap occurs both in the Yap1-1 isoform, which has a single WW domain, and in the Yap1-2 isoform, which has tandem WW domains [44]. In contrast, vertebrate TAZ occurs almost exclusively in an isoform with a single WW domain [45]. Recently, a second TAZ isoform was identified in medaka that possesses tandem WW domains like the Yap1-2 isoform [45]. In this study, the affinity between TAZ and PPXY-containing ligands was enhanced by the presence of the additional WW domain, potentially affecting partner protein selection. However, it remains to be determined whether the second TAZ isoform shares binding partners and functional redundancy with the Yap1-2 isoform during early fish development.

Our studies have demonstrated that active Yap can repress retinal photoreceptor cell differentiation, at least in part, by directly blocking the Rx transcriptional machinery. However, the upstream factors that control the timing of Hippo-Yap activation remain unknown. It is possible that the apicobasal polarity protein Crumbs (CRB) is a candidate upstream sensor regulating Yap activity during retinogenesis. Pellissier *et al*. have recently reported that the loss of both CRB1 and CRB2 during early retinogenesis in mice prevents the development of a separate photoreceptor layer and leads to a loss of retinal function that is reminiscent of the abnormalities of humans with Leber Congenital Amaurosis [46]. Pellissier *et al*. also showed that the transcription of *connective tissue growth factor,* a Yap-regulated gene, was reduced in CRB1/CRB2 double KO mice [46], suggesting a critical role for CRB in regulating Yap activity and RPC proliferation during vertebrate retinogenesis. Other cell-extrinsic signals, such as mechanical forces, GPCR ligands, cell density, and serum concentration, have been shown to regulate the Hippo pathway during tissue-specific stem cell differentiation [47]. Understanding exactly how such a variety of microenvironmental signals might coordinate Hippo pathway signaling during RPC/photoreceptor cell fate determination awaits future study.

## Materials and Methods

### Statement on the Ethical Treatment of Animals

This study was carried out in strict accordance with the recommendations in the ethical guidelines of Tokyo Medical and Dental University. All experimental protocols in this study were approved by the Animal Welfare Committee of Tokyo Medical and Dental University (Permit Number: 2010-212C). All experiments were performed in a manner that minimized pain and discomfort.

### Zebrafish Maintenance and Staging

The TL wild type (WT) strain was maintained essentially as described in “The Zebrafish Book” [48]. Embryos were produced by natural matings and staged by standard morphological criteria or by hours or days post-fertilization (hpf or dpf), as described [49].

### Phylogenetic Tree

Amino acid sequences of Mst1 and Mst2 of various species were obtained from the Ensembl database. The Ensembl ID numbers of the sequences used were as follows: human MST1 (ENSP00000361892), mouse MST1 (ENSMUSP00000018353), *Xenopus* Mst1 (ENSXETP00000049383), medaka Mst1 (ENSORLP00000024937), pufferfish Mst1 (ENSTNIP00000007894), stickleback Mst1 (ENSGACP00000000023), human MST2 (ENSP00000390500), mouse MST2 (ENSMUSP00000018476), *Xenopus* Mst2 (ENSXETP00000038688), zebrafish Mst2 (ENSDARP00000015367), medaka Mst2 (ENSORLP00000023002), pufferfish Mst2 (ENSTNIP00000012004), stickleback Mst2 (ENSGACP00000004790) and *Drosophila* Hippo (FBpp0085688). A Genescan prediction from the Ensembl database was used to obtain the complete medaka Mst2 sequence. These amino acid sequences were aligned with each other and any positions containing gaps were eliminated. The phylogenetic tree was constructed using the neighbor-joining method and ClustalX software [50]. The reliability of the tree was estimated using the bootstrap method and 10,000 replications.

### Antisense Morpholino (MO) against *mst2*

The *mst2* MO (5′-ATGGG CTGTT AAAAC ACAAT GAGGA-3′) was designed to target the splice acceptor site of exon 4 of the zebrafish *mst2* gene (ENSDARG00000011312) and was synthesized by GeneTools, LLC (Philomath, OR). For knockdown, *mst2* MO solution (13.3 or 20 ng) was injected into the yolks of one-cell to four-cell stage zebrafish embryos immediately beneath the cell body. The standard negative control MO (5′-CCTCT TACCT CAGTT ACAAT TTATA-3′) was injected into a control cohort of zebrafish embryos in a similar fashion. Reduction in *mst2* mRNA was confirmed by RT-PCR analysis using the oligonucleotide primer pair 5′-AGCCA TTCAC AAGGA ATCAG G-3′ and 5′-GGTAA GTTGT CCAGC TACTC C-3′.

### Total RNA Extraction and RT-PCR Analysis

Total RNA was isolated from 7–10 zebrafish embryos at 2 dpf using TRIzol reagent according to the manufacturer’s protocol (Invitrogen). First-strand cDNA was synthesized from 1 µg total RNA using SuperscriptIII reverse transcriptase (Invitrogen) and oligo-dT primer. Primers used for RT-PCR analysis of mRNA expression in zebrafish extracts were as follows: for *rhodopsin*, 5′-ACAGA GGGAC CGGCA TTCTA CG-3′ and 5′-CAGGC CATGA CCCAG GTGAA G-3′; for *crx*, 5′-AGAGA CGCGG CCGTC CCAAG-3′ and 5′-TCTTC ACGCA TCTTT CCTTC C-3′; for *otx5*, 5′-ACCCT AACAC TCCAC GGAAA C-3′ and 5′-TGCAG TCCAG GCCTG TAAAG-3′; for *otx2*, 5′-ATGAT GTCGT ATCTC AAGCA ACC-3′ and 5′-AGGAA GTGGA ACCAG CATAG CC-3′; for *rx1*, 5′-GATGC CGACA TGTTC TCCAA C-3′ and 5′-CGCCA TGGGC TGCAT GCTTT G-3′; for *rx2*, 5′-GGCTG CCTCT CCACA GAAAG-3′ and 5′-AAACC ACACC TGAAC TCGAA C-3′; for *β-actin*, 5′-CAGCT TCACC ACCAC AGC-3′ and 5′-GTGGA TACCG CAAGA TTCC-3′.

### Plasmid Construction

Because our laboratory has been studying the small fish medaka for decades, we took advantage of the availability of medaka *yap (WT)* cDNA and the evolutionary conservation of *yap* sequences among fish species to create plasmids expressing mutated *yap* cDNAs. Our full-length medaka *yap (WT)* cDNA was originally isolated as a homolog of the human *YAP1-2β* isoform [19], [44]. We subcloned this cDNA into a modified pCS2+ vector, which positions the FLAG tag at the N-terminus of the insert. The *Yap (5SA)* mutant and its variants with point mutations or deleted domains were generated by the inverse PCR-based method using the primers listed in Table S1. For heat shock experiments, *yap (WT)*, *yap (5SA)* and its variants were cloned into a modified pCS2+ vector in which the CMV promoter was replaced with the zebrafish *hsp70* promoter and the EGFP coding sequence (see Fig. 4A). For retina-specific expression, the zebrafish *hsp70* promoter was replaced with a 4-kb fragment of the medaka *rx3* promoter, which was isolated by PCR using the medaka genome and the primer pair 5′-CCGCC GGCCT CTGAT GTGAT GTTGA CAAA-3′ and 5′-CCCCA TGGTT GTCTA AAAAG GAACT TAAA-3′ (see Fig. 5A) [51]. For co-immunoprecipitation analyses, the PCR-amplified full-length zebrafish *rx1* cDNA was cloned into a pMyc-CMV5 vector (the kind gift of Dr. T. Katada, University of Tokyo), placing the Myc tag at the N-terminus of the insert. The *Rx1* variant in which the PPXY motif was deleted was generated by the inverse PCR-based method using the primers listed in Table S1.

### Synthesis of Capped mRNA for Microinjection

Capped sense strand mRNA was synthesized using SP6 RNA polymerase and the mMESSAGE mMACHINE system (Ambion) according to the manufacturer’s protocol. RNA injections were performed as described previously [52].

### Microarray Analysis

TRIzol reagent was used to extract total RNA at 42, 48 or 54 hpf from whole zebrafish embryos that had been injected with *yap (WT)* or *yap (5SA)* mRNA. RNA quality assurance, cDNA synthesis, and cRNA labeling and hybridization were carried out by Takara Bio Inc. (Otsu, Japan) using a Zebrafish (V3) Gene Expression Microarray 4X44K, the Low Input Quick Amp Labeling Kit, the Gene Expression Hybridization Kit, and the Gene Expression Wash Buffers Pack (all from Agilent Technologies) according to the manufacturer’s protocols. Raw data extraction and analyses were performed using Agilent Feature Extraction software (Agilent Technologies). Gene Ontology analysis was conducted using KeyMolnet software (IMMD Inc., Tokyo, Japan).

### Antibodies

Mouse monoclonal anti-FLAG (F1804) and rabbit polyclonal anti-Myc (C3956) antibodies (Abs) were purchased from Sigma–Aldrich Co.

### Co-immunoprecipitation Assay

Co-immunoprecipitation assays were performed as previously described [53], with some modifications. HEK293T cells were plated in 10-cm dishes and transfected with the appropriate expression plasmids as described in the Figure Legends. Cells were washed twice with phosphate-buffered saline (PBS) and homogenized in binding buffer [150 mM NaCl, 1 mM EDTA, 0.5% Nonidet P-40, 1 mM EGTA, 5% glycerol, and 20 mM Tris-HCl (pH 7.4)] supplemented with 4 µg/mL aprotinin, 50 mM NaF, and 0.1 mM Na_3_VO_4_. Extracts were clarified by centrifugation for 10 min at 15,000g, and supernatants were precleared by incubation with 20 µl protein G-agarose beads (GE Healthcare) for 1 h at 4°C. After preclearing, supernatants were incubated with 20 µl anti-FLAG M2-agarose beads (Sigma–Aldrich) overnight at 4°C. The beads were washed three times with binding buffer, boiled in SDS sample buffer, and centrifuged. The supernatants were fractionated by SDS-PAGE and analyzed by Western blotting as described below.

### Western Blotting

Immunoprecipitated materials and total cell extracts obtained as described above were fractionated by SDS-PAGE and transferred electrophoretically to PVDF membranes. Membranes were incubated in blocking solution [2% nonfat skim milk in Tris-buffered saline (TBS)] for 1 h at room temperature (RT). Blocked membranes were incubated with anti-FLAG or anti-Myc Ab in 5% BSA/TBS overnight at 4°C. Membranes were washed three times in 0.2% Tween 20 in TBS (TBST), incubated with anti-mouse/rabbit horseradish peroxidase-conjugated Abs in 2% nonfat skim milk in TBS for 1 h followed by three washes in TBST. Proteins were visualized using the SuperSignal West Femto Kit (Pierce) and a ChemiDoc XRS system (Bio-Rad), as described [52].

## Supporting Information

Figure S1 **Knockdown analysis of the zebrafish** ***mst2*** **gene.** (A) Alignment of amino acid sequence of zebrafish Mst2 with its human and mouse homologs. Amino acids were aligned using the ClustalX program. Residues are colored according to their physicochemical properties [54]. Gaps have been introduced to optimize alignment. *, critical autophosphorylation site reflecting kinase activation [55]. Black underline, SARAH domain. Arrow, insertion site of the in-frame stop codon in the zebrafish *mst2* morphant. (B) Phylogenetic tree inferred from amino acid sequences of Mst proteins. Statistical significance (%) is shown on each node. Nodes with closed circles represent species divergences, while the node with the open circle represents gene duplication. Scale bar, 0.02 substitutions per site. (C) Top panel, schematic illustration of the target site of the *mst2* MO. Arrows indicate positions of primer pairs used in RT-PCR evaluation of MO efficacy. Bottom panel, partial sequences of native and intron 3-inserted *mst2* mRNAs. The stop codon (in red lettering) occurs in the inserted intron 3 of *mst2* mRNA, resulting in the production of a truncated Mst2 protein. (D) RT-PCR validation of *mst2* MO efficacy. Total RNA was extracted at 52 hpf from embryos injected with control MO (20 ng) or *mst2* MO (13.3 ng) and showing the phenotypes of abnormal eye pigmentation plus short body length (AP & SL), or abnormal eye morphology (AM) plus AP & SL. β-actin, loading control.
(TIF)

Figure S2 **Morphological analysis of** ***yap (5SA)*** **mRNA-injected zebrafish embryos during the gastrulation and segmentation periods.** (A) Alignment of amino acid sequence of medaka Yap with its zebrafish homolog performed as in Fig. S1. *, conserved serine residues phosphorylated by Lats. (B) Representative images of *yap (5SA) mRNA*-injected zebrafish embryos (N = 3) at the indicated developmental stages during gastrulation. Embryos were injected with EGFP mRNA as a control. (C) Representative lateral images of the embryos in (B) examined at the indicated stages during segmentation.
(TIF)

Figure S3 **Reduced retinal gene expression in** ***mst2*** **morphants.** (A) RT-PCR analysis of mRNA levels of the indicated retinal genes in zebrafish embryos injected with control MO or *mst2* MO and examined at 52 hpf. *Mst2* morphants were grouped by abnormal phenotype, as indicated. (B) RT-PCR analysis of *rhodopsin* mRNA expression in the morphants in (A). For A and B, results are representative of two independent trials.
(TIF)

Figure S4 **A proposed model for the dual function of Hippo-Yap signaling during retinal progenitor cell proliferation versus photoreceptor cell differentiation.** Left panel: When the Hippo pathway is inactive, activated Yap transactivates cell proliferation-related genes via association with TEAD. At the same time, activated Yap represses Rx1-mediated transcription of the *otx, crx* and *rhodopsin* genes, which results in suppression of photoreceptor cell differentiation. Right panel: When the Hippo pathway is active, Yap activation is blocked. TEAD on its own is insufficient to drive cell proliferation-related gene transcription. Without Yap-mediated suppression, Rx1-mediated transcription of *otx, crx* and *rhodopsin* is upregulated, leading to the differentiation of mature photoreceptor cells.
(TIF)

Figure S5 **The PPXY motif in retinal transcription factors is highly conserved among vertebrate species.** Sequence alignment of C-terminal amino acid residues of the retinal TFs Rx (A), ROR (B) and NRL (C) from the indicated species. Residues are colored according to their physicochemical properties. The red boxes indicate the positions of the PPXY motif. The blue boxes indicate the OAR domain of Rx (transactivation domain), the α-Helix10 domain of ROR, and the leucine zipper of NRL.
(TIF)

Table S1 **List of primer sequences for plasmid constructions.**
(TIF)

Methods S1 **Supporting methods.**
(DOC)
