# Supplementary figures and images for: The Hippo Pathway Controls a Switch between Retinal Progenitor Cell Proliferation and Photoreceptor Cell Differentiation in Zebrafish
Source: PLoS One. 2014 May 14;9(5):e97365. doi: 10.1371/journal.pone.0097365 (PMC4020862; doi:10.1371/journal.pone.0097365)

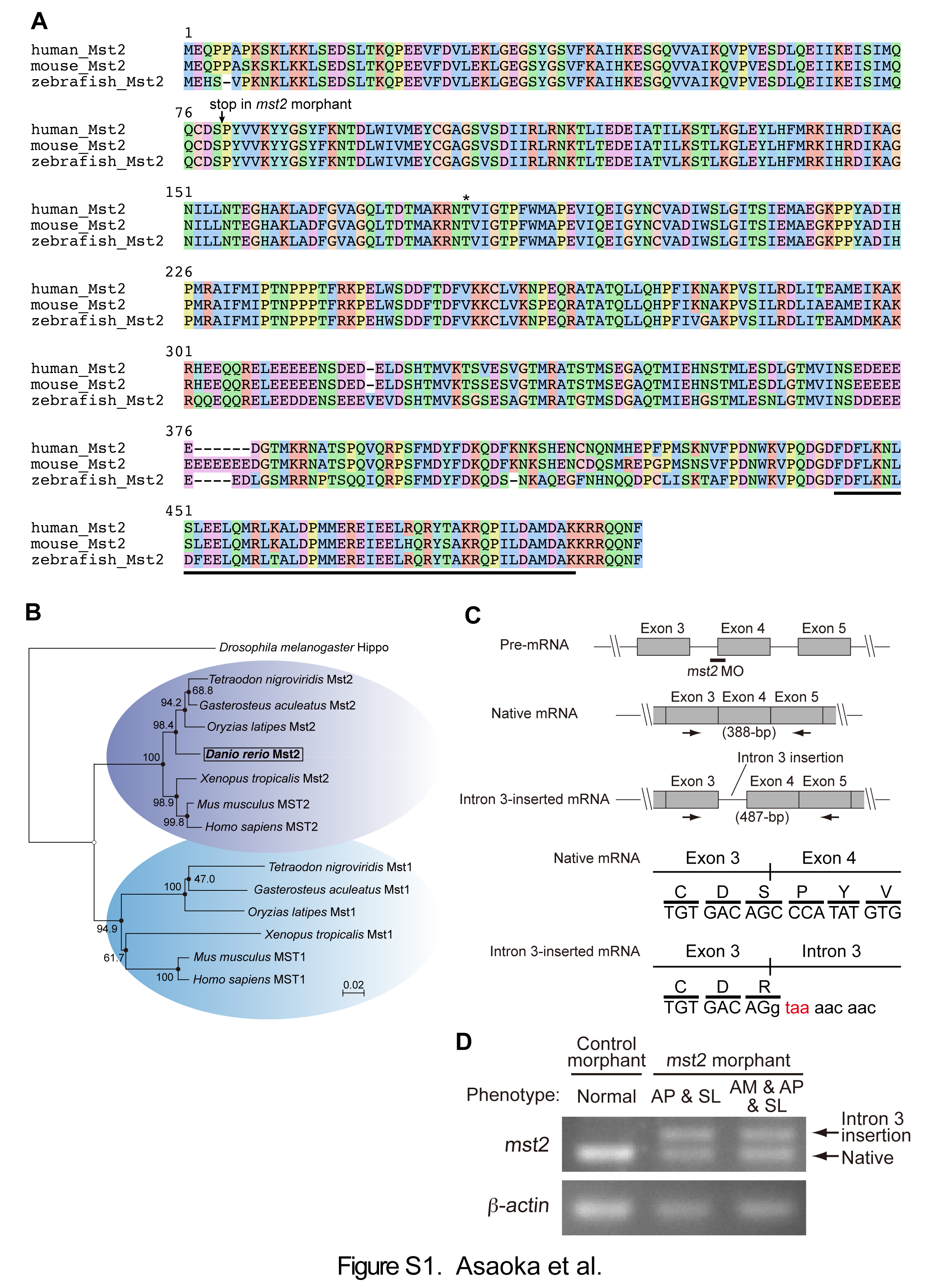

Supplement: Figure S1 — Knockdown analysis of the zebrafish mst2 gene. (A) Alignment of amino acid sequence of zebrafish Mst2 with its human and mouse homologs. Amino acids were aligned using the ClustalX program. Residues are colored according to their physicochemical properties [54]. Gaps have been introduced to optimize alignment. *, critical autophosphorylation site reflecting kinase activation [55]. Black underline, SARAH domain. Arrow, insertion site of the in-frame stop codon in the zebrafish mst2 morphant. (B) Phylogenetic tree inferred from amino acid sequences of Mst proteins. Statistical significance (%) is shown on each node. Nodes with closed circles represent species divergences, while the node with the open circle represents gene duplication. Scale bar, 0.02 substitutions per site. (C) Top panel, schematic illustration of the target site of the mst2 MO. Arrows indicate positions of primer pairs used in RT-PCR evaluation of MO efficacy. Bottom panel, partial sequences of native and intron 3-inserted mst2 mRNAs. The stop codon (in red lettering) occurs in the inserted intron 3 of mst2 mRNA, resulting in the production of a truncated Mst2 protein. (D) RT-PCR validation of mst2 MO efficacy. Total RNA was extracted at 52 hpf from embryos injected with control MO (20 ng) or mst2 MO (13.3 ng) and showing the phenotypes of abnormal eye pigmentation plus short body length (AP & SL), or abnormal eye morphology (AM) plus AP & SL. β-actin, loading control. (TIF) [file pone.0097365.s001.tif]

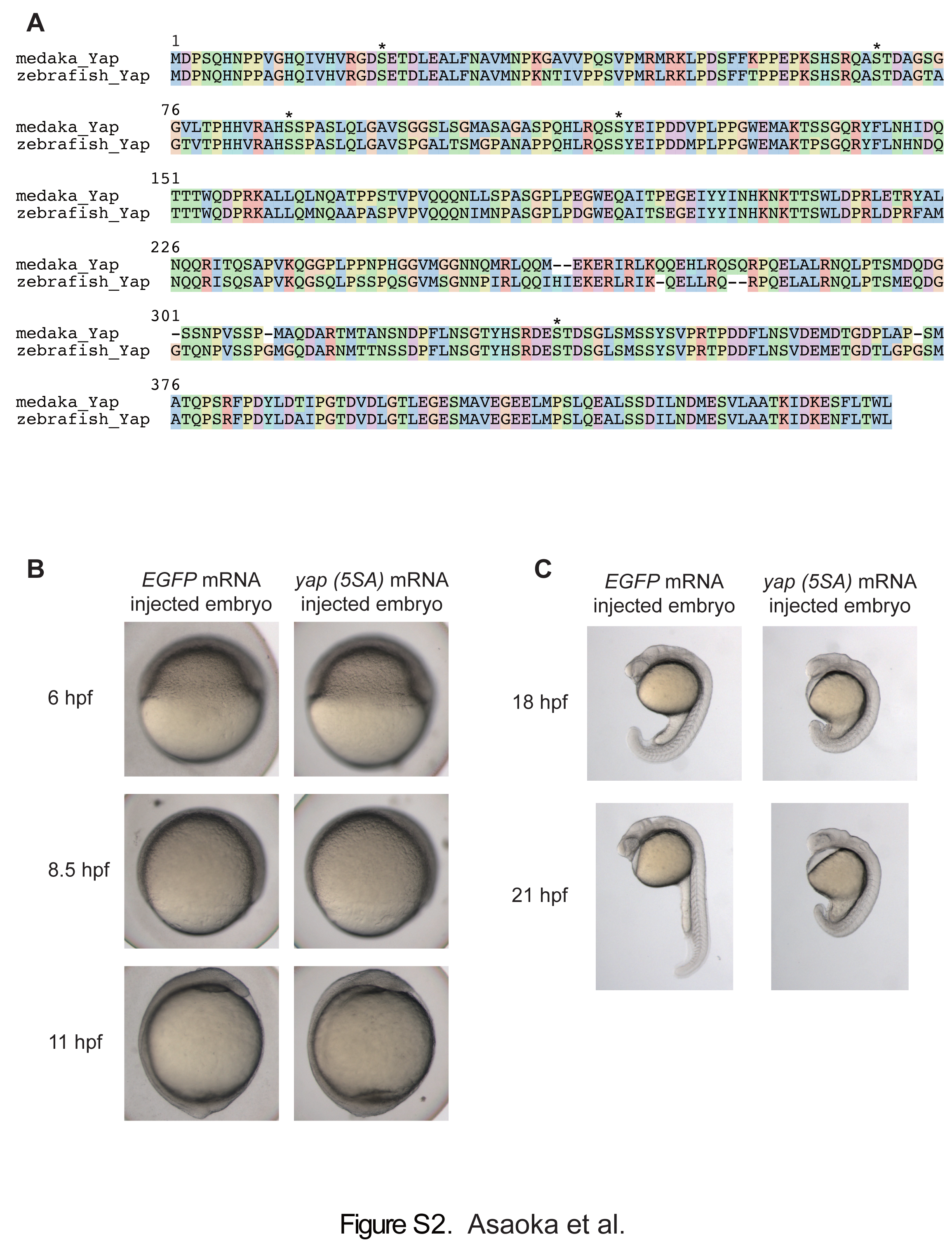

Supplement: Figure S2 — Morphological analysis of yap (5SA) mRNA-injected zebrafish embryos during the gastrulation and segmentation periods. (A) Alignment of amino acid sequence of medaka Yap with its zebrafish homolog performed as in Fig. S1. *, conserved serine residues phosphorylated by Lats. (B) Representative images of yap (5SA) mRNA-injected zebrafish embryos (N = 3) at the indicated developmental stages during gastrulation. Embryos were injected with EGFP mRNA as a control. (C) Representative lateral images of the embryos in (B) examined at the indicated stages during segmentation. (TIF) [file pone.0097365.s002.tif]

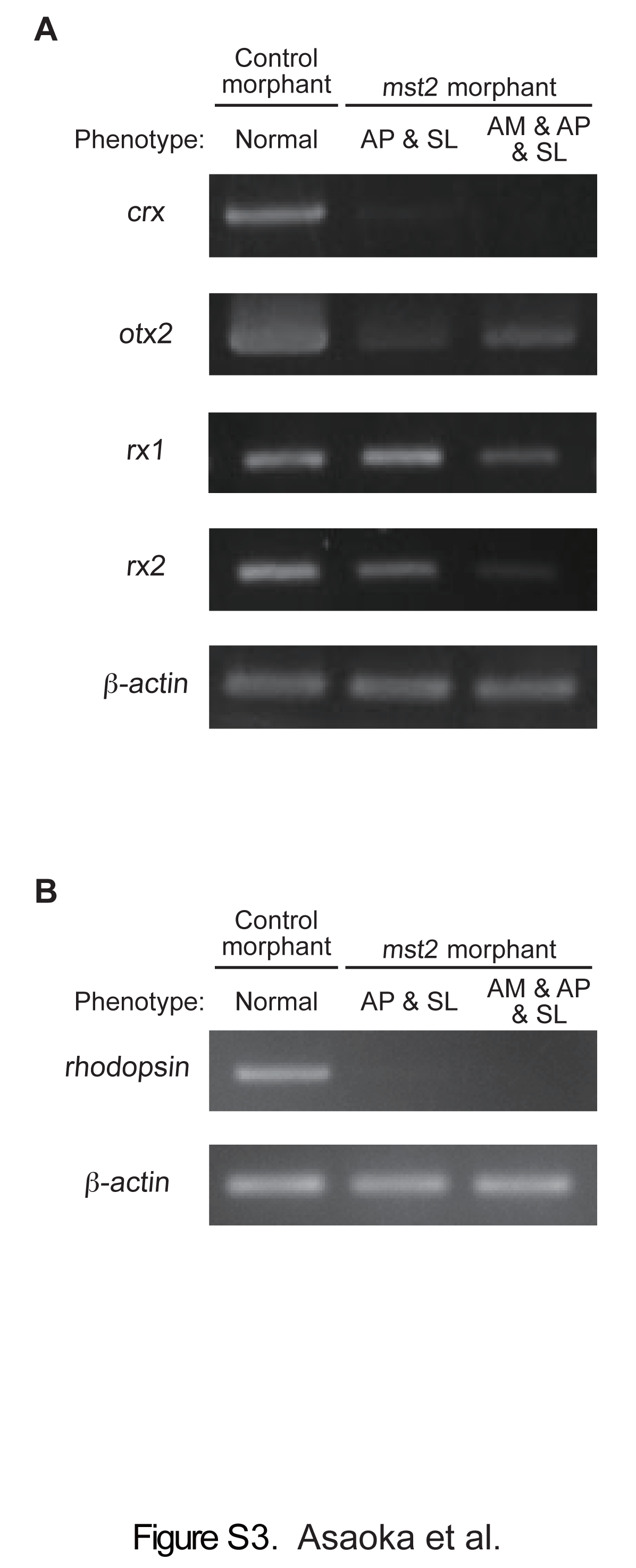

Supplement: Figure S3 — Reduced retinal gene expression in mst2 morphants. (A) RT-PCR analysis of mRNA levels of the indicated retinal genes in zebrafish embryos injected with control MO or mst2 MO and examined at 52 hpf. Mst2 morphants were grouped by abnormal phenotype, as indicated. (B) RT-PCR analysis of rhodopsin mRNA expression in the morphants in (A). For A and B, results are representative of two independent trials. (TIF) [file pone.0097365.s003.tif]

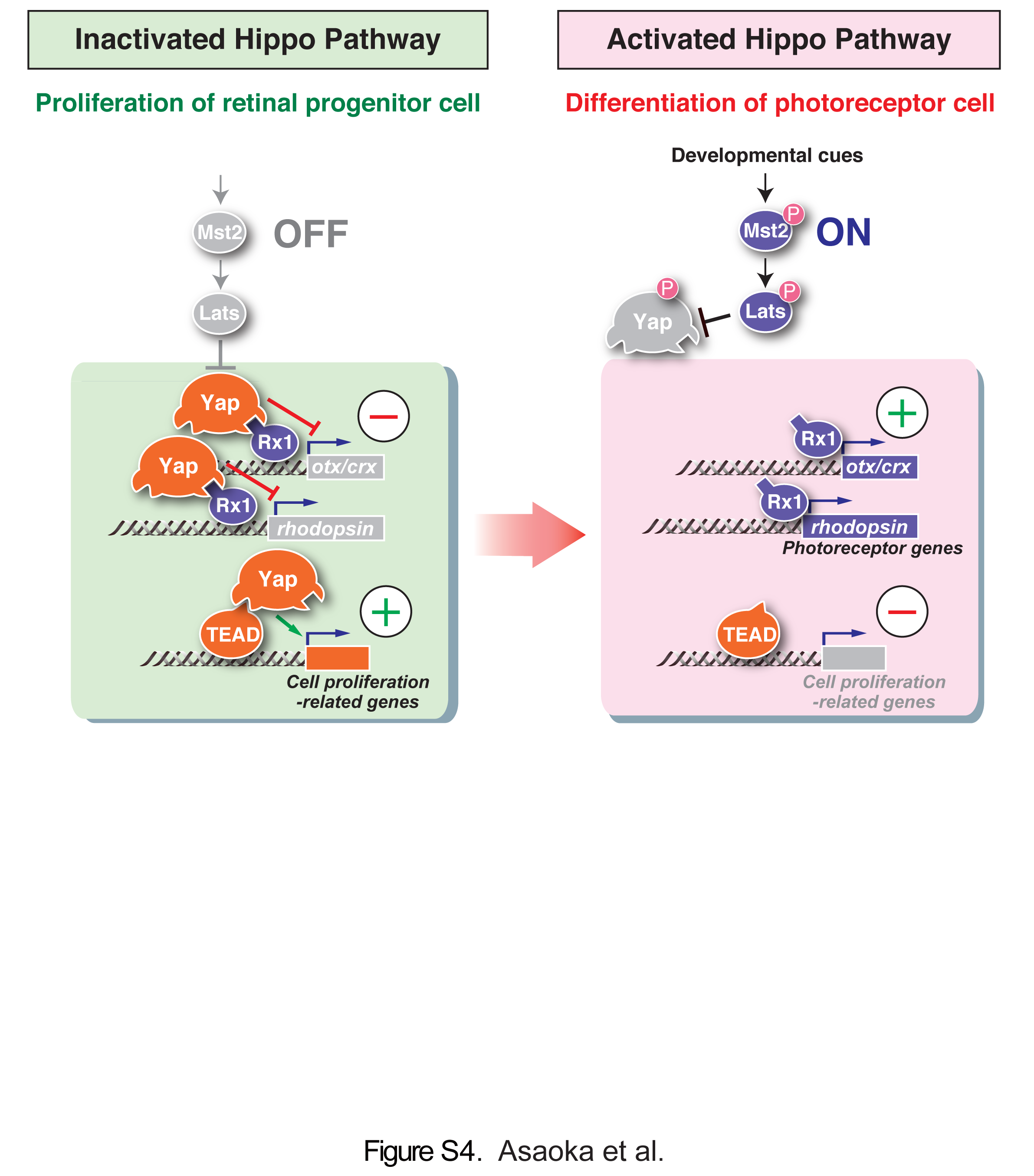

Supplement: Figure S4 — A proposed model for the dual function of Hippo-Yap signaling during retinal progenitor cell proliferation versus photoreceptor cell differentiation. Left panel: When the Hippo pathway is inactive, activated Yap transactivates cell proliferation-related genes via association with TEAD. At the same time, activated Yap represses Rx1-mediated transcription of the otx, crx and rhodopsin genes, which results in suppression of photoreceptor cell differentiation. Right panel: When the Hippo pathway is active, Yap activation is blocked. TEAD on its own is insufficient to drive cell proliferation-related gene transcription. Without Yap-mediated suppression, Rx1-mediated transcription of otx, crx and rhodopsin is upregulated, leading to the differentiation of mature photoreceptor cells. (TIF) [file pone.0097365.s004.tif]

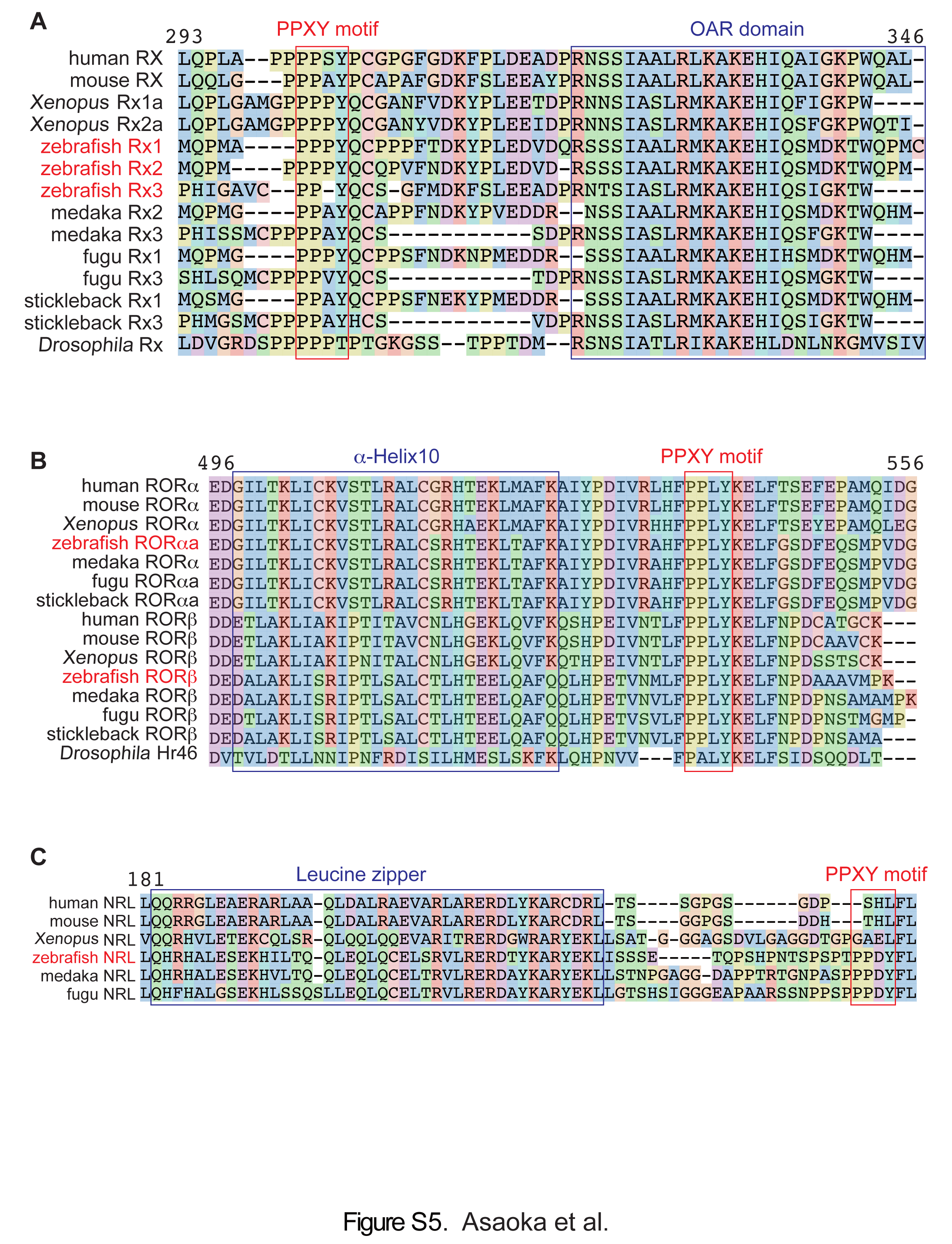

Supplement: Figure S5 — The PPXY motif in retinal transcription factors is highly conserved among vertebrate species. Sequence alignment of C-terminal amino acid residues of the retinal TFs Rx (A), ROR (B) and NRL (C) from the indicated species. Residues are colored according to their physicochemical properties. The red boxes indicate the positions of the PPXY motif. The blue boxes indicate the OAR domain of Rx (transactivation domain), the α-Helix10 domain of ROR, and the leucine zipper of NRL. (TIF) [file pone.0097365.s005.tif]

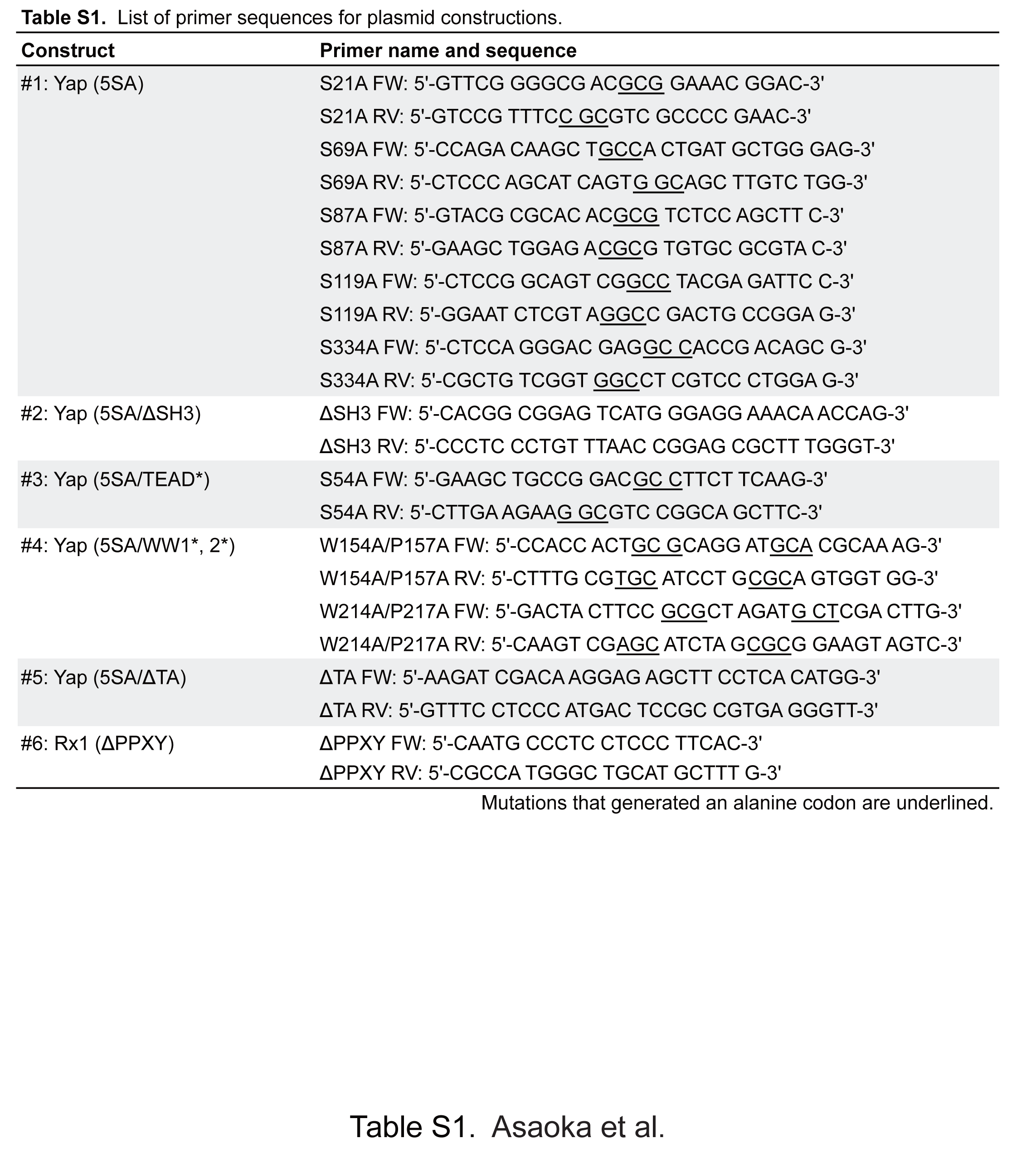

Supplement: Table S1 — List of primer sequences for plasmid constructions. (TIF) [file pone.0097365.s006.tif]
