# Supplementary material for: The Hippo Pathway Controls a Switch between Retinal Progenitor Cell Proliferation and Photoreceptor Cell Differentiation in Zebrafish
Source: PLoS One. 2014 May 14;9(5):e97365. doi: 10.1371/journal.pone.0097365 (PMC4020862; doi:10.1371/journal.pone.0097365)
Supplement: Methods S1 — Supporting methods. (DOC) [file pone.0097365.s007.doc]

**ONLINE SUPPLEMENTAL MATERIAL**

**Methods S1**

**Sequence alignment of multiple retinal transcription factors across species**

Amino acid sequences of Rx, ROR and NRL proteins of various species were obtained from the Ensembl or GenBank databases. The Ensembl IDs or GenBank accession numbers of the sequences used were as follows: human Rx (ENSP00000334813), mouse Rx (ENSMUSP00000025396), *Xenopus* Rx1a (AF001048), *Xenopus* Rx2a (AF001049), zebrafish Rx1 (ENSDARP00000096944), zebrafish Rx2 (ENSDARP00000105437), zebrafish Rx3 (ENSDARP00000069319), medaka Rx2 (ENSORLP00000022547), medaka Rx3 (ENSORLP00000007573), fugu Rx1 (ENSTRUP00000023915), fugu Rx3 (ENSTRUP00000031282), stickleback Rx1 (ENSGACP00000022326), stickleback Rx3 (ENSGACP00000021721), *Drosophila* Rx (FBpp0071447), human RORα (ENSP00000261523), human RORβ (ENSP00000379507), mouse RORα (ENSMUSP00000034766), mouse RORβ (ENSMUSP00000047597), *Xenopus* RORα (ENSXETP00000045670), *Xenopus* RORβ (ENSXETP00000045673), zebrafish RORαa (ENSDARP00000106236), zebrafish RORβ (ENSDARP00000084181), medaka RORα (ENSORLP00000009581), medaka RORβ (ENSORLP00000015578), fugu RORαa (ENSTNIP00000011225), fugu RORβ (ENSTNIP00000017712), stickleback RORαa (ENSGACP00000014125), stickleback RORβ (ENSGACP00000015289), *Drosophila* Hr46 (FBpp0291630), human NRL (ENSP00000454062), mouse NRL (ENSMUSP00000107035), *Xenopus* NRL (ENSXETP00000047873), zebrafish NRL (ENSDARP00000064615), medaka NRL (ENSORLP00000002498), and fugu NRL (ENSTNIP00000020783). Amino acid sequence alignments were performed using the ClustalX program.
